# Supplementary material for: Are carbon emissions trading and green financial instruments synergistic? -Comprehensive quantitative research based on content analysis
Source: PLoS One. 2024 Mar 7;19(3):e0298601. doi: 10.1371/journal.pone.0298601 (PMC10919601; doi:10.1371/journal.pone.0298601)
Supplement: S1 Data — (ZIP) [file pone.0298601.s001.zip › Data/地级市Z_F工作报告.zip!/║■▒▒/╛ú├┼╩╨/└┤╫╘╣1⁄2╓┌║┼í╛┬φ┐╦╩2╛▌═°í┐_2019.docx]

各位代表：

　　现在，我代表市人民政府向大会报告工作，请予审议，并请市政协各位委员和列席人员提出意见。

　　一、2018年工作回顾

　　刚刚过去的一年，是荆门发展史上不平凡的一年，是直面挑战、迎难而上、砥砺前行的一年。面对经济下行压力加大、困难矛盾增多、改革发展稳定任务繁重的宏观环境，我们坚持以习近平新时代中国特色社会主义思想为指导,在省委、省政府和市委的坚强领导下,在市人大和市政协的监督支持下，认真贯彻落实习近平总书记视察湖北重要讲话精神，紧紧围绕全面建设湖北区域性增长极的战略定位，统筹推进稳增长、促改革、调结构、惠民生、防风险各项工作，经济社会持续健康发展，较好地完成了市九届人大二次会议确定的目标任务。一年来，我们主要做了以下工作：

　　（一）着力抓谋划重实施，“稳”的基础更加牢固。围绕抓重点、补短板、强弱项，强化顶层设计和制度安排。聚焦实体经济发展，修订完善招商引资政策，构建加强现有企业培育政策体系，树立崇商、安商、富商的鲜明导向。聚焦产业、区域协调发展，制定支持现代服务业、现代物流业、文化产业、外贸产业发展的政策措施，坚持全要素、全产业链与全供应链、全地域推进县域经济发展。聚焦高质量发展，出台加快新旧动能转换实施意见，大力发展20个重点产业，进一步夯实了发展基础。全年实现地区生产总值1847.89亿元，增长8%；社会消费品零售总额增长12.3%，居全省第2位；固定资产投资增长11.5%，居全省第3位；地方一般公共预算收入增长12%，居全省第4位；规模以上工业增加值增长8.3%；实际利用外资增长9.8%；出口总额增长9%；城镇常住居民人均可支配收入33779元，增长7.86%；农村常住居民人均可支配收入18776元，增长9.37%；居民消费价格涨幅2.4%；城镇登记失业率3.49%；人口自然增长率3.78‰；完成省定节能减排目标。

　　（二）着力抓投资强实体，“进”的动能更加强劲。深入推进产业链招商，引进嘉士利华中生产基地、万诺线路板、申通电商物流科技产业园、海特生物高端原料药等539个亿元以上项目。完善“三级包联”服务和项目落地机制，实行双月集中开工、季度分类拉练，全市在建亿元以上项目1058个，同比增加70个，其中，玲珑轮胎、技联志成智慧电能制造、奥美医疗、科顺高端防水材料等514个亿元以上项目新开工建设；三雷拖拉机整机、亿纬锂能高性能锂锰电池、弘润特种汽车玻璃原片、永创鑫电路板等369个亿元以上项目建成投产。招商引资实际到位资金1288.4亿元，增长20.4%。开通“企业服务110”，推出服务企业用工“十大福利”，兑现惠企政策资金50亿元，降低企业综合成本60亿元，全市新增规模以上工业企业80家、达到1113家。

　　（三）着力抓转型调结构，“新”的特色更加彰显。完成工业技改投资304.6亿元，增长14.3%；实施兼并重组项目51个。电子信息、新能源新材料、再生资源利用与环保、装备制造产业产值分别增长35.4%、34.6%、26.4%、18%；“荆门产”猎豹CS9汽车下线，鲲龙AG600在漳河机场水上首飞。高新技术产业增加值增长12.7%，国家技术转移中部中心荆门分中心挂牌，荆门高新区获批国家创新型特色园区，在国家级高新区排名第63位，两年跃升20位。划定粮食生产功能区和重要农产品保护区711万亩，粮食总产295.2万吨，农产品加工业产值与农业总产值之比提高到3.5∶1，新型农业经营主体达到2.2万个。钟祥蝉联全省“三农”发展成绩突出首强县（市），沙洋再次入围全省十强。启动中心城区现代都市农业示范圈建设，持续推进荆钟、荆京、荆沙三条乡村振兴示范带建设，漳河爱飞客小镇、京山网球小镇等特色小镇初具雏形。服务业增加值增长10.5%，网上销售额增长68.6%。2018荆门爱飞客飞行大会游客突破30万人次，圣境山滑翔基地成为国家航空飞行营地，京山网球节、沙洋油菜花节等品牌效应进一步放大。

　　（四）着力抓改革促开放，“好”的因素更加集聚。供给侧结构性改革扎实推进，关停所有9万吨以下煤矿，关闭石膏矿12家，封闭矿山井口25个。“互联网+放管服”改革深入实施，启动“最多跑一次”改革，公布“马上办、网上办、就近办、一次办”清单，推进公共资源全流程电子化交易，在全省率先实施公共服务“两集中”改革、实现省市县乡村政务服务事项“一网通办”，完成26条政务热线整合。投融资体制机制改革取得新突破，开展银企“手拉手”活动，设立政银合作基金6.57亿元，发行企业债券7只66亿元；一批非法集资案件得到有效处置，商业性不良贷款率压减至2.53%；累计落地PPP项目40个，我市被国务院表彰为全国推广PPP模式成效明显市。农村综合改革成效明显，我市被确定为全国农村集体产权制度改革整体推进试点市，钟祥三产融合经验入选全国百佳案例。全力扩大对外开放，荆门国际内陆港、保税物流中心（B型）建设加快推进，开通“荆门——武汉”通勤航线及汉江首条集装箱定班航线，荆门海关获批成立。

　　（五）着力抓环保优生态，“绿”的底色更加靓丽。加快国家“双修”试点城市建设，编制荆门2049远景发展战略规划，推进东宝山、象山和竹皮河“两山一河”改造升级，象山景区三个出入口基本建成，竹皮河全段截污干管通水运行；实行招商引资环境准入“负面清单”制度，科学划定工业园区开发边界。打好蓝天、碧水、净土保卫战，淘汰黄标车11116辆，关停中心城区20蒸吨/小时以下燃煤锅炉；全面消除“国考”断面劣五类水体，县级以上集中式饮用水源地水质达标率100%；中国农谷核心区土壤环境保护优先示范项目、胡双磷地区土壤污染治理与修复试点项目进展顺利。大力实施“四个三重大生态工程”，在全省率先实现乡镇污水处理设施全覆盖；建改农村户厕17.5万户、城乡公厕120个；荆门静脉产业园一期项目全面实施，新建乡镇垃圾转运站49座；精准灭荒4.12万亩。全省“四个三重大生态工程”、城市建设绿色发展现场会在我市召开，钟祥大力推进生态工程建设、打造宜居乡村做法受到国务院表彰。

　　（六）着力抓民生增福祉，“惠”的广度更加拓展。民生改善三年行动圆满收官，去年年初确定的民生实事全部办结。出台脱贫攻坚“十二条”，实施特色扶贫产业项目5590个，完成易地搬迁2286人，改造危房和土坯房2784户，募集健康扶贫补充医疗救助基金8760万元，全年实现36个贫困村出列、22718名贫困人口脱贫。组织就业创业培训22259人，发放创业担保贷款20772万元，扶持创业6120人，新增城镇就业50121人。深化长期护理保险改革试点工作，基本养老保险、基本医疗保险参保率分别达到89.1%、92%。新建7个城市社区居家养老服务中心、48个农村老年人互助照料中心、13所留守儿童服务站、186个“希望家园”。完成农村义务教育学校“全面改薄”和39所义务教育学校标准化改造，我市成为全省唯一的全国教育信息化项目试点地区，屈家岭金色农谷青少年教育基地、漳河爱飞客小镇分别被确定为全国中小学生研学实践教育营地、基地。开展紧密型医联体建设试点，分级诊疗制度进一步完善。“一村一名大学生村医”培养计划启动实施。有效应对“问题疫苗”事件，全市未发生重大传染病疫情。建成九渊书吧20家，农民体育健身工程实现全覆盖，屈家岭国家考古遗址公园博物馆主体工程完工。中心城区南北汽车客运新站竣工，即将投入使用。吹响创建全国文明城市“集结号”，荆门城市形象持续提升。推进新型综合社区建设，中心城区1000平方米以上的社区服务综合体达到26个，120个城市社区形成“三社联动”工作格局，1065个村（社区）实行积分制管理，完成村（社区）“两委”换届选举工作。积极创建国家社会信用体系建设示范城市，我市在全国市州信用监测中排名第10位。扎实开展扫黑除恶专项斗争，打掉了一批涉黑涉恶团伙和黑恶势力“保护伞”。加强突发事件应急处置，持续开展安全生产隐患大排查大整治、打非治违专项行动，全力保障食品药品安全，有效防控非洲猪瘟，社会大局和谐稳定，人民群众获得感、幸福感和安全感进一步增强。

　　与此同时，我们进一步加强国防教育、人民防空、民兵预备役、双拥共建、退役军人服务管理等工作，支持驻荆部队深化改革，军民深度融合发展取得新进步。妇女儿童、青少年、老年人、残疾人权益保障得到新加强，民族、宗教、外事、侨务、对台、统计、住房公积金、机关事务、新闻出版、保密、地方志、档案、科普、气象、移民、防震减灾、对口援建等工作再上新台阶。

　　（七）着力抓作风提效能，“干”的氛围更加浓厚。一年来，我们旗帜鲜明讲政治，始终在政治立场、政治方向、政治原则、政治道路上同以习近平同志为核心的党中央保持高度一致。坚持不懈提能力，按照“一般干部应知应会、中层干部行家里手、领导干部专家权威”的要求，开展政府系统大学习、大培训。坚定不移重法治，进一步完善政府工作规则、议事决策程序，向市人大提交地方性法规议案《荆门市建筑装饰装修管理条例》《荆门市农业面源污染防治条例》，出台政府规章《荆门市临街建筑外立面管理办法》，办理人大代表建议218件、政协委员提案378件。驰而不息正风纪，开展“整顿干部作风、整治发展环境”活动，坚决纠正隐形变异的“四风”问题，切实加强审计监督，严格防控政府廉政风险，推动从严治党主体责任落地落实。

　　各位代表，事非经过不知难，看似寻常最奇崛。我们的每一点成绩、每一次进步，都是在省委、省政府坚强领导、亲切关怀下取得的，是在市委总揽全局、科学决策下取得的，是在市人大、市政协有效监督、大力支持下取得的，是在全市人民勠力同心、砥砺奋进中取得的。在此，我代表市人民政府，向全市人民，向人大代表、政协委员，向各民主党派、工商联、人民团体、无党派人士，向离退休老领导、老同志，向驻荆部队、武警官兵，向所有关心支持荆门发展的社会各界朋友，致以崇高的敬意和衷心的感谢!

　　各位代表，安不忘危，兴不忘忧。我们也清醒地看到，我市经济社会发展还存在诸多问题，政府工作还存在许多不足，主要是：动能转换与高质量发展的要求还不适应，产业结构不优，新经济势强力弱，现代服务业发展比较滞后；城市规划建设管理水平与现代化精致城市的要求还不适应，中心城区能级和品位不高，交通物流短板明显；民生保障与基本公共服务均等化、普惠化、便捷化的要求还不适应，环境保护欠账较多，生态建设任重道远，公共服务配套不足，社会治理还需进一步加强；干部能力作风与建设法治政府、服务型政府的要求还不适应，营商环境有待进一步优化，工作质效不高、担当作为不够、攻坚克难办法不多等问题还不同程度地存在。我们将始终坚持问题导向，勇于刀刃向内、自我革命，以不回避、不遮掩、不懈怠的态度，切实加以解决。

　　二、2019年工作安排

　　2019年，是新中国成立70周年的大庆之年，是全面建成小康社会的关键之年，是实现高质量发展的大考之年。做好今年的工作，我们必须登高望远、审时度势，既要正视中美经贸摩擦等外部不确定因素可能产生的不利影响，更要看到我国发展拥有足够的韧性、巨大的潜力，坚定办好自己事情的信心和决心；既要直面经济运行稳中有变、变中有忧的挑战，更要明确经济长期向好的态势不会改变，全面用好重要战略机遇期；既要认清控债务、严监管、强环保等多重叠加影响，更要把握国家强化逆周期调节的新形势，抢抓各种政策密集出台、政策环境进一步宽松的“风口”；既要立足荆门市情和发展阶段性特征进行科学谋划，更要对接国家和省重大战略，主动融入“一芯驱动、两带支撑、三区协同”高质量发展区域和产业战略布局。只要我们始终保持“不畏浮云遮望眼”“千磨万击还坚劲”的战略定力，坚持变中求新、变中求进、变中突破，就一定能够奋力谱写新时代荆门高质量发展新篇章。

　　今年政府工作的总体要求是：以习近平新时代中国特色社会主义思想为指导，全面贯彻党的十九大、十九届二中、三中全会精神，认真落实中央和全省经济工作会议、省十三届人大二次会议部署，坚持稳中求进工作总基调，坚持“六稳”工作要求，坚持高质量发展，落实“一芯驱动、两带支撑、三区协同”区域和产业战略布局，打好三大攻坚战，加快推进产业转型升级，深入实施乡村振兴战略，着力建设现代化精致城市，全面深化改革开放，持续优化营商环境，切实保障和改善民生，奋力争当江汉平原振兴发展示范区排头兵，为全面建成小康社会收官奠定决定性基础，以优异成绩庆祝新中国成立70周年。

　　今年工作的主要预期目标是：地区生产总值增长8%左右；规模以上工业增加值增长8%左右；固定资产投资增长11%；社会消费品零售总额增长11%；地方一般公共预算收入增长8%；进出口总额增长8%；居民消费价格涨幅控制在省定目标以内；城镇登记失业率控制在4.5%以内；人口自然增长率5‰左右；城乡常住居民人均可支配收入增幅与经济增长基本同步；节能减排完成省定目标。重点做好以下工作：

　　（一）全力构建现代化产业体系

　　深化供给侧结构性改革，落实20个重点产业培育行动方案，推动产业集群化、产品高新化、模式新型化、业态多样化、品牌高端化。

　　推动制造业高质量发展。实施“百企技改”行动，完成工业技改投资340亿元。实施产业链垂直整合行动，以磷化工、农产品加工企业为重点，完成兼并重组项目30个。实施战略性新兴产业倍增行动，大力发展高端装备制造、再生资源利用与环保、新能源新材料、电子信息、生物医药、大健康等产业，高质量建设国家通用航空产业综合示范区，力争战略性新兴产业产值增长20%。实施军民融合发展行动，加快荆门航空产业园军民融合产业示范基地、康沁药业国家军特药西南生产基地建设。实施骨干企业培育行动，支持荆门石化转型发展，支持格林美、新洋丰、凯龙化工、京山轻机等龙头企业稳产增产扩产、延链强链补链，支持76家细分领域“隐形冠军”做大做强。实施质量提升行动，创建全国质量强市示范城市，在规模以上工业企业推行全面质量管理体系。

　　推动现代服务业突破性发展。高标准建设省级服务业综合示范区，打造爱飞客航空、农耕文化旅游、养生山水等品牌。推进龙山中央商务区等项目建设，做大做强现代商贸业；实施荆门国际内陆港公铁物流中心、沙洋新港物流园等项目，建设全国重要的区域性物流基地；加大湖北群艺、文化宫等文化创意产业园区（街区）建设力度，积极培育新兴服务业态；编制全域旅游规划，加快漳河爱飞客小镇、莫愁村、圣境山旅游综合体等项目建设，办好首届湖北油菜花节，打造航空运动与长寿养生旅游目的地、产旅融合型全域旅游示范区。

　　推动新经济新业态蓬勃发展。聚焦数字经济、智能经济、绿色经济、创意经济、流量经济、共享经济，构建新经济产业生态圈。加快人工智能与产业发展融合，重点推动10家企业开展智能化改造。不断拓展大数据应用空间，在生产经营、精准医疗、城市管理等领域实施一批示范项目，实现规模以上企业“上云”全覆盖。完善电子商务网络体系，加强电子商务质量监管，全市规模以上企业电子商务应用普及率达到90%。

　　（二）全力加快新旧动能转换

　　突出发展第一要务、创新第一动力、人才第一资源，不断提升经济含金量、含新量、含绿量。

　　进一步突出创新引领。积极建设国家创新型城市，力争高新技术产业增加值增长12.5%，研究与试验发展经费占GDP比重达到2.3%，高新技术、科技型中小企业分别达到280家、300家。构建以企业为主体、市场为导向、主导产业为重点的区域创新体系，组建省级产业技术研究院，新增省级以上科技创新平台10家。

　　进一步激活人才资源。柔性引进15个重大产业人才项目、10个博士团队，定向培养800名技能人才，做实院士专家工作站，新增工作站10家。加快科创社区建设，提供产学研居娱一体化服务。全面落实人才政策，让各类人才充分施展才干、聪明才智迸发涌流。

　　进一步优化投资结构。完善项目储备库、建设库、达效库，从投资源头改造旧动能、培育新引擎。统筹推进950个亿元以上项目建设，开工建设新宙邦电解液、润都制药、君健新材料等重点项目，加快东方雨虹环保防水材料、众星汽车零部件、弘信柔性电子等重点项目建设，推动盈德气体、万华生态板、新视野机床等重点项目投产达效。

　　进一步夯实县域支撑。支持每个县（市、区）重点培育1-2个主导产业，每个主导产业重点培育3-4家龙头企业。统筹建设20个特色工业园区，推动产业集群集聚发展。抓好国家新型城镇化综合试点工作，加快培育一批商贸大镇、旅游名镇、工业强镇。支持钟祥在全国县域经济百强中争先进位，支持沙洋、京山、东宝建设经济强县。

　　（三）全力防范和化解重大风险

　　坚持从最坏处准备，向最好处努力，牢牢把握工作主动权，坚决守住不发生区域性系统性风险底线。

　　严格防控金融风险。严厉打击非法集资等违法违规金融活动，强化互联网金融监管，完善类金融机构监管体系，规范发展地方交易场所。通过“八个一批”有效化解银行不良贷款，确保全市商业性不良贷款率控制在2.5%以内。开展恶意逃废债等专项整治，营造良好的金融生态。引导企业强化风险意识，优化债务结构，降低债务杠杆率。

　　严格防控债务风险。处理好防风险与促发展的关系，加强政府债务限额和预算管理，严肃查处违规融资和担保行为。积极争取一般债券、专项债券额度，进一步规范政府与社会资本合作。坚决遏制政府隐性债务增量，积极稳妥化解存量。分类推进融资平台公司市场化转型。

　　严格防控社会风险。按照配套设施现代化、生活环境宜居化、社会服务集成化要求，加快新型综合社区建设。加强社会治安综合治理，严厉打击违法犯罪活动，深入推进扫黑除恶专项斗争，提升平安荆门建设水平。做好“七五”普法、信访、人民调解、公共法律服务、网络空间治理等工作，坚持和发展新时代“枫桥经验”。严格落实安全生产责任制，深化安全生产领域改革，坚决杜绝重特大安全事故发生。加强应急处突工作，提升防灾减灾救灾能力。严守食药品从生产到销售的每一道防线，全力保障群众吃上安全食品、用上放心药。

　　（四）全力打好精准脱贫攻坚战

　　以一鼓作气、攻城拔寨的决心，打好打赢脱贫攻坚战，确保全面小康路上不落一人。

　　坚持打好扶贫“组合拳”。大力实施“九大扶贫攻坚行动”，下足绣花功夫推进精准脱贫。聚焦产业扶贫，构建贫困户与市场主体利益紧密联结机制、收入稳定增长机制；抓好“四位一体”健康扶贫保障政策落地，充分发挥补充医疗救助基金作用，切实防止因病致贫返贫；完成贫困户土坯房改造任务，加快易地搬迁配套设施建设，确保搬得出、稳得住、能致富；严格落实保障兜底政策措施，实现低保政策与精准扶贫有效衔接；进一步加强贫困村“五基”建设，不断改善生产生活条件。

　　坚持激发脱贫内生动力。把扶贫与扶志相结合，建立生产奖补、以工代赈、劳务奖补等正向激励机制，引导贫困群众劳动增收、就业创业致富，避免养“懒汉”。把扶贫与扶智相结合，加大贫困家庭子女教育资助力度，防止因贫失学辍学，阻断贫困代际传递。把扶贫与扶能相结合，大力开展技能培训，增强可持续发展能力。

　　坚持脱真贫真脱贫。聚焦“两不愁三保障”，实行最严格的考核评估制度，加强扶贫成效考核督查和脱贫退出验收，做到脱贫工作务实、脱贫过程扎实、脱贫结果真实。健全防范返贫机制，做实脱贫后续工作，对已脱贫人口在脱贫攻坚期内不脱政策。加大对收入水平略高于建档立卡贫困户群体的帮扶力度。保证现行标准下的脱贫质量，实现剩余的49个贫困村全部出列、37186名贫困人口全部脱贫。

　　（五）全力加强生态环境保护和治理

　　牢固树立“两山”理念，统筹山水林田湖草系统治理，让荆门天更蓝、山更绿、水更清、空气更清新。

　　全面抓好突出环境问题整治。巩固中央环保督察“回头看”、省级督查反馈意见整改成果，深入实施污染防治攻坚三年行动计划。打好“双十”工程这场硬仗，以壮士断腕、背水一战的决心抓好沿汉江化工企业关改搬转等工作，让群众共享清水绿岸、鱼翔浅底的美好环境。严格执行“河湖长制”，抓好汉江、天门河、竹皮河、长湖、浰河等重点流域综合整治，加强国控断面所在流域污染防控，实现流域水质整体持续好转。统筹推进控煤、控车、控尘、控烧、控工业污染，力争全市空气质量优良天数比例达到80%，让老百姓出门少戴口罩、多见蓝天。

　　全面加强生态修复和环境保护。坚持生态优先、绿色发展，保障“一带两屏四网六廊”生态安全，坚守绿水青山。打造“四个三重大生态工程”全省样板，完成精准灭荒1.6万亩，确保乡镇污水处理设施稳定运行、达标排放，全域推广农村生活污水无动力或微动力处理，推行“户分类、组保洁、村收集、镇转运、县处理”生活垃圾处理模式，建设439省道江山至石牌段绿色廊道。加强农业面源污染防治，实现“一控两减三基本”目标。加大地质灾害防治力度，持续推进水土保持、矿山地质环境恢复等生态项目建设。

　　全面推进绿色低碳循环发展。深化国家循环经济示范市建设，依托特色循环产业园，深入推进园区循环化改造，着力打造石化、磷化、绿色建材、再生资源回收利用等循环经济链条，实施循环经济项目25个，完成投资20亿元以上。推进绿色技术开发利用，加快节能环保、清洁生产、清洁能源等产业发展。强化能源、水资源、建设用地等消耗总量和开发强度双控，完善生态补偿机制，加强领导干部自然资源资产任中、离任审计。

　　（六）全力实施乡村振兴战略

　　落实产业兴旺、生态宜居、乡风文明、治理有效、生活富裕的总要求，促进农业全面升级、农村全面进步、农民全面发展，在“中国农谷”建设上实现新作为、新突破。

　　扎实推进农业现代化。坚持结构调整强农，大力发展优质稻米等8个重点农业产业，推广生态高效种养模式160万亩。坚持质量品牌强农，打响以“中国农谷·长寿荆门”为内涵的“荆品名门”区域公共品牌，新认证“三品一标”30个，农产品质量安全例行抽检合格率达到98%。坚持绿色发展强农，创建农牧结合生态养殖示范场40家、水产健康养殖示范场10家，农作物秸秆综合利用率达到95%，畜禽养殖废弃物综合利用率达到80%。坚持科技创新强农，农机总动力达到480万千瓦，主要农作物耕种收综合机械化水平达到81%，创建国家农业高新技术产业示范区。坚持三产融合强农，加快建设中心城区现代都市农业示范圈；高标准打造京山桥米和食用菌、钟祥长寿食品、沙洋和掇刀高油酸油菜、东宝绿色家居、屈家岭生态农产品、漳河果冻橙和清水小龙虾产业园。坚持人才振兴强农，深入实施“三乡工程”，鼓励市场主体和各类人才投身乡村建设；积极培育新型职业农民和新型农业经营主体，发展多种形式适度规模经营，推动小农户与大生产、大市场有机衔接。

　　扎实推进农村现代化。新建高标准农田71万亩，新增高效节水灌溉面积2万亩。加快鄂北水资源配置二期工程（荆门部分）前期工作，推进碾盘山水利水电枢纽工程建设，抓好汉江一二期堤防和长湖湖堤加固、汉北河（荆门段）防洪综合治理、钱家河等6条中小河流治理工程建设，对68座小型水库进行除险加固。依托荆钟、荆京、荆沙三条示范带，突出产业集聚型、创新创业型、文化传承型、强农富农型，分别打造1个以上特色小镇或田园综合体。统筹推进小城镇、村庄人居环境综合整治，加快美丽宜居乡村建设。

　　扎实推进乡村治理体系和治理能力现代化。健全和创新村党组织领导的充满活力的村民自治机制。大力发展村级集体经济，力争每村集体经济年收入达到5万元以上。实施“领头雁”计划，打造一支懂农业、爱农村、爱农民的工作队伍。加强传统村落保护和自然风貌管控，传承历史文脉，留住乡愁记忆。培育文明乡风、良好家风、淳朴民风。

　　（七）全力打造现代化精致城市

　　坚持走内涵式发展道路，统筹城市规划建设管理，加快补齐城市能级和品位短板。

　　不断提升综合承载能力。强化规划引领和刚性约束作用，启动中心城区国土空间规划体系前期研究工作；编制高铁新站等重点片区控制性详细规划、城市设计和交通专项规划；推进掇刀石大道以南等片区控制性详细规划修改。实施中心城区重点城建项目130个。完成泉水大道建设和金龙泉大道改造；加快荆东大道改造、龙井大道南段、航空路、尉迟恭路、华科路东段、罗汉山隧道、漳河环库公路等建设；基本消除城市黑臭水体，全面完成象山景区改造，抓好东宝山改造工程；实施原葛洲坝水泥厂山体修复治理；配套完善停车场等公共服务设施；确保荆门传媒中心、“两馆两中心”、市民中心等重大公用工程投入使用。

　　不断提升精细管理水平。有序推动专业市场退城进园，构建长效管理机制。持续开展市容市貌提升工程，抓好深度保洁，加强街巷常态化管理，实施违规占道、大货车进城、建筑渣土抛撒、噪音油烟污染等专项整治。加快静脉产业园建设，扩大生活垃圾分类试点。即查即处新增违建，加快消化存量违建。

　　不断提升城市文明程度。大力实施“八大提升行动”，举全市之力抓好全国文明城市创建。抓好历史文化街区和文化遗产保护，让文化深度融入城市发展。推进社会主义核心价值观教育、“书香荆门”建设，总结提炼荆门精神，弘扬主旋律，倡导新风尚。

　　（八）全力推进改革开放

　　坚持改革不停顿、开放不止步，不断注入发展新动力、厚植发展新优势。

　　切实完成政府机构改革任务。科学设置、全面优化政府机构及职能配置，进一步转变政府职能，破解机构重叠、职责交叉、权责脱节等问题，构建简约高效的行政管理体制。

　　抓好农村综合改革。深化农村承包土地“三权分置”改革，探索宅基地“三权分置”改革。全面完成农村集体产权制度改革任务。扎实推进农业水价综合改革、供销合作社改革和五三农场企业化改革。

　　加快投融资体制机制改革。抓好上市后备企业培育、辅导，力争1-2家企业报会。加强与泰康人寿等大型保险集团合作，推动险资入实。加大金融产品创新力度，大力发展普惠金融、绿色金融、科技金融。

　　统筹推进其他领域改革。深化国企国资改革，完成国有企业“三供一业”分离移交任务，推进市级国有企业混合所有制改革，积极化解国企改制遗留问题。完成社保征管体制改革任务。坚持预算和绩效管理一体化，提高财政资源配置效率和使用效益。深入推进城管执法体制改革和城市管理领域市场化改革。

　　打造内陆开放新高地。配合推动沿江高铁武荆宜段开工建设，确保蒙华铁路荆门段投入运营、枣潜高速通车。积极争取冷水机场实行军民合用。提升漳河机场和汉江荆门组合港通航能力。加快荆门国际内陆港建设、保税物流中心（B型）运营。抓好荆门海关保障建设。加强外贸主体培育，引导外贸企业拓展多元化市场。

　　（九）全力营造良好营商环境

　　坚持把创优营商环境作为应对困难挑战的重器，把民营企业和民营企业家当成自己人，打造便捷高效、稳定透明、公平竞争的营商环境。

　　以更大力度深化“放管服”改革。深化“互联网+放管服”改革，加强数字政府建设，全面推行“一网一门一次”改革，提升实体和网上办事大厅“一站式”服务能力，市县两级政务服务事项网上可办率、一窗受理率、“四办”政务服务事项覆盖率达到80%，让数据多跑路、群众少跑腿、办事像“网购”一样方便。实行集成极速审批，推广一般工业项目“先建后验”改革经验，实施“标准地”出让和“一事一办”“证明直通车”改革，开展并联审批、容缺审批、多评合一、联审联验，进一步压缩企业开办和建设工程项目审批时限。加强中介组织清理整顿和市场培育，建好用好“中介超市”，加大政府购买中介服务力度。坚持一窗受理、流程简便、快审快兑，常态化抓好惠企政策兑现。

　　以更大力度支持民营经济发展。构建“亲清”政商关系，深入开展“千名干部联千家非公企业交千名非公经济人士朋友”行动。全面落实国家减税降费政策，修订《涉企行政事业性收费目录清单》，清单之外一律不收费，清单收费一律按下限执行。实行市场准入负面清单，打破各种“卷帘门”“玻璃门”“旋转门”。规范涉企执法行为，杜绝“一刀切”“一关了之”“一律不批”。构建政银企深度对接机制，扩大政银合作、银担合作融资增信规模，积极争取政策性救助基金支持，缓解企业融资难融资贵问题。清理低效闲置土地，充分保障重点企业、重点项目用地需求。开展政校企对接，推行企业新型学徒制培训，共建实训基地，定向培训人才。支持企业参与电力直接交易，力争交易量占全市工业用电量的50%以上。清理规范供气服务收费，降低企业用气成本。切实保障民营企业合法经营，保护民营企业家人身和财产安全。

　　以更大力度推进招商引资。紧盯七大主导产业，突出产业细分领域，建设目标数据库、项目数据库、合作载体库，开展定向精准招商，引进投资10亿元以上项目30个以上、5亿元以上项目100个以上。进一步创新产业链招商、以商招商、委托招商、股权招商、基金公司招商，加强对注册率、开工率、资金到位率、投产率考核，确保招商引资质量。

　　（十）全力保障和改善民生

　　坚持决策以民生为首要、支出以民生为首位、工作以民生为首事，既尽力而为，又量力而行，不断满足人民日益增长的美好生活需要。

　　大力提高就业创业水平。加快发展人力资源服务业，打造“网上就业局”，落实稳就业、保用工政策措施。加强创业孵化基地建设，扶持创业5000人。做好城镇登记失业人员、高校毕业生、去产能职工、农村转移劳动力、被征地农民、退役军人等重点群体就业工作。开展和谐劳动关系创建活动，保障农民工工资及时足额发放，维护劳动者合法权益。

　　大力健全社会保障体系。实施“社保共享”计划，实现城乡居民应保尽保。完善长期护理保险制度和服务体系。落实城乡低保和特困供养制度，提高临时救助、医疗救助标准。加强对农村“三留守”人员、残疾人等弱势群体帮扶。加快构建以居家为基础、社区为依托、机构为补充、医养相结合、城乡全覆盖的养老服务体系。

　　大力办好人民满意的教育。坚持教育优先发展，提升学前教育质效；切实解决中小学生课外负担重、“择校热”、“大班额”等突出问题，规范校外培训机构；优化高中资源配置，加快“新高考”改革；积极发展职业教育，深化产教融合、校企合作，确保荆门职业学院新增在校生1500人；支持荆楚理工学院特色办学，为地方发展输送高素质人才。

　　大力推进“健康荆门”建设。实施“健康荆门2030”行动计划，健全分级诊疗制度，建设市县两级紧密型医疗联合体，推进城市优质医疗资源共享下沉。建立现代医院管理制度，持续深化公立医院综合改革。加强疫苗预防接种管理和宣传引导，抓好重大疾病防治。加强“全面两孩”配套服务，促进人口均衡发展。积极发展康复事业。支持中医药传承创新发展。

　　大力发展文体事业。加快建设中心城区“15分钟文体活动圈”，建成市博物馆新馆，完成乡镇（街道）综合文化站提档升级和村级文化广场建设任务。广泛开展全民健身活动，扎实推进“一县一品”文体项目建设，筹办好2019亚洲跳伞锦标赛、“中国农谷杯”全国传统弓射箭比赛、中国（京山）绿林网球英雄会、市第十届老年人健身大会等一批精品体育赛事。

　　加强国防教育和国防后备力量建设，推动军民深度融合发展，扎实做好“双拥”工作，巩固军政军民团结的良好局面。继续做好外事、侨务、民族、宗教、援疆援恩、对台、统计、保密、档案、地方志、物价、移民、机关事务等工作，发挥好工会、共青团、妇联、社科联、科协、侨联、工商联、文联、残联等群团组织的重要作用。

　　各位代表，民有所呼，必有所应。保障和改善民生要一年接着一年干、一件接着一件办。今年我们将继续实施一批民生实事：

　　1.新增城镇就业40000人，组织就业创业技能培训26000人，发放创业担保贷款1.8亿元。

　　2.新开工保障性安居工程3000套，基本建成3000套，分配入住3000套。完成农村危房改造2500户。

　　3.新改建10所城乡公办幼儿园，启动中心城区天鹅学校、长宁新城实验学校建设和望兵石学校、名泉小学扩建工程。

　　4.继续实施“一村一名大学生村医”培养计划，免费培养200名临床专业全日制大专学历乡村医生。

　　5.对10000例疑似肺结核病患者进行免费筛查，对500例艾滋病病毒感染者和病人实施免费抗病毒治疗，对20000名城乡已婚适龄妇女开展“两癌”免费检查。

　　6.举办文化惠民活动1600场次，放映农村公益电影16000场次。

　　7.在中心城区全面推行惠民农贸超市建设，在中心城区农贸市场全面建立农药残留快检室。

　　8.建成中心城区车站社区、关公社区等20个社区服务综合体；完成中心城区15条背街小巷刷黑改造。

　　9.新改建农村公路500公里、农村电网配电台区434个。解决10万农村居民饮水安全问题。

　　10.改造农村无害化厕所12万所，新改建城市公厕40座。

　　各位代表，我们的一切工作都是为了人民，我们将始终和群众站在一起、想在一起、干在一起，以百姓忧为忧、以百姓苦为苦、以百姓乐为乐，多办顺民心的实事、得民心的好事、暖民心的难事，努力让群众生活更安康、让人生出彩的舞台更宽广、让每个人实现梦想的道路更通畅！

　　三、进一步加强政府自身建设

　　各位代表，加强政府自身建设永远在路上，只有进行时，没有完成时。我们将以永不懈怠的精神状态和一往无前的奋斗姿态，深化拓展“两整”活动，不断提升政府治理能力和水平，努力建设新时代人民满意的法治政府、服务型政府。

　　坚持忠诚为政，增强政府凝聚力。把坚持党的领导贯穿政府工作全过程、各方面，树牢“四个意识”，坚定“四个自信”，践行“两个维护”，坚定不移地贯彻落实党中央国务院大政方针，推动省委省政府及市委决策部署落地落细。

　　坚持以学促政，增强政府创新力。按照“既要政治过硬，也要本领高强”的要求，加快建设学习型政府，全面增强“八种本领”。注重提升专业能力，加快补齐能力弱项、本领短板，掌握“金刚钻”、干好“瓷器活”。注重坚持问题导向，聚焦发展中不平衡不充分问题，精准施策、靶向发力，提高政策的针对性、实施的有效性。注重推动改革创新，以前瞻眼光、全局视野、创新思维谋划推动工作，努力做到采它山之玉、补自身之短、创荆门之新。

　　坚持依法行政，增强政府公信力。把政府一切工作纳入法治轨道，以法治立规矩、以法治立秩序、以法治立诚信、以法治立质量。自觉接受市人大及其常委会法律监督、工作监督和市政协民主监督，诚恳听取各民主党派、工商联、无党派人士、人民团体和社会各界的意见。严格执行科学民主依法决策程序，深化行政执法体制改革，促进严格规范公正文明执法。

　　坚持实干勤政，增强政府执行力。大力倡导“智圆行简、实心实政”的工作作风，在战略部署上“扣扣子”，在责任履行上“担担子”，在任务落实上“钉钉子”。坚持真抓实干，撸起袖子、甩开膀子、扑下身子，干在发展一线、闯在改革前线、冲在攻坚火线。坚持责任上肩，强化督办检查，打通责任落实的“最后一米”。坚持为民履职，面对面、心贴心、实打实做好群众工作，让发展业绩更实、惠民答卷更优，使群众感到政府离自己很近、与自己很亲。

　　坚持从严治政，增强政府免疫力。政府党组率先垂范、以上带下，压实全面从严治党主体责任，做到真管真严、敢管敢严、长管长严。锲而不舍落实中央八项规定实施细则精神和省、市实施办法，坚决反对形式主义、官僚主义，坚决反对特权思想、特权现象，严防“四风”回潮复燃。健全土地出让、工程建设、产权交易、政府采购等制度，加强公共资金、公共资源、国有资产监管，实现审计监督全覆盖，扎牢织密制度笼子。全力支持纪委监委工作，有腐必反、有贪必肃，保证干部清正、政府清廉、政治清明。

　　各位代表，幸福都是奋斗出来的，只有干出来的精彩，没有等出来的辉煌。让我们更加紧密地团结在以习近平同志为核心的党中央周围，在省委、省政府和市委的坚强领导下，紧紧依靠全市人民，不驰于空想、不鹜于虚声，埋头苦干、真抓实干，为推动高质量发展、决胜全面建成小康社会而努力奋斗！
